# Algorithms, allyship, and advice: A qualitative analysis of fertility tracker marketing

**DOI:** 10.1177/20552076251356395

**Published:** 2025-08-10

**Authors:** Kate Sheridan Clay, Sue Ziebland, John Powell

**Affiliations:** Nuffield Department of Primary Care Health Sciences, 6396 Oxford University, Oxford, UK

**Keywords:** Digital health, infertility, reproductive health

## Abstract

**Objective:**

Proponents of ‘Femtech’, digital technology targeting women, frame them as instruments of women's empowerment that will revolutionise digital care. Its critics argue industry uses the moniker to popularise platforms that surveil reproductive data for profit. This qualitative analysis critically examines the marketing language used to promote digital interventions for managing infertility and discusses implications for users.

**Methods:**

We use an inductive thematic analysis approach to assess advertising for 15 top fertility tracking applications. Using both Foucauldian critical theory and feminist theory, we identified a code set and major themes connecting marketing content to broader rhetoric around (in)fertility, gender equality, and power dynamics in health care.

**Results:**

The main themes identified are: an emphasis on technological rather than human intelligence, allyship, online safety, and reliable advice. Reliance on non-human support is emphasised across multiple themes, and the framing of contested issues such as privacy and security is explored after the introduction of anti-abortion legislation in the United States, where many of the platform companies and users are based.

**Conclusion:**

We demonstrate how company marketing encourages users to centre digital tracking technologies in their fertility journeys. In doing so, Femtech marketers place the complex burden of reproductive labour on women's shoulders while offering a digital reprieve (for a fee).

## Introduction

Approximately one in six couples globally struggle with infertility, defined as the inability to conceive after 12 months of regular intercourse.^1^ Though causes of infertility are split between cisgender heterosexual men and women nearly equally, the burden of treatment and management is a reproductive labour placed overwhelmingly on women.^2^ The experience of infertility is a laborious one; treatment interventions have mental and physical side effects, many women feel isolated and stigmatised, and few employment protections exist to assist working women with time off for appointments or to recover from miscarriages and treatment side effects.^3–5^ Given these pressures and constraints, it is unsurprising that a digital wave of support has emerged offering education, products, and resources to women with infertility. Online communities offer free advice, comfort, and camaraderie, as well as the option for anonymity.^6^ Digital health platforms have changed the way women can approach fertility treatment by offering home access to hormone testing, high-tech fertility tracking, educational resources, and telehealth services with medical professionals. Though mobile apps are limited in the services they can provide compared to a clinic, they offer minimally invasive, affordable, anonymous sources of education, information gathering, and support networks, all of which can be reached quickly and simply. These innovations fall under the umbrella of ‘Femtech’, or software, products, and services focused on using technology to support women's health.^7^

Despite representing one of the most popular Femtech app categories, the ways these trackers present themselves to users and the public through their marketing remains unstudied. The internet and social media platforms have long been a principal source of information for people seeking to understand and get support for their fertility challenges.^8,9^ As people trying to conceive turn to the internet for guidance and resources, and use of tracking apps continues to grow globally, it's important to carefully analyse the kinds of marketing and claims patients may be consuming, and consider the impact it may have on care-seeking and health behaviours.^10^ This analysis critically examines the marketing language to explore how contemporary technology designed for reproductive bodies and fertility frame themselves to consumers, and the implications of the digitisation of fertility tracking for women. It examines complexity and tensions across app advertising messaging to explore how such content may shape societal views towards female reproductive health and provides insights into the diffusion of digital fertility trackers into mainstream health technologies. It considers implications of this language for users and clinicians, and discusses the thematic findings within the context of significant sociotechnical shifts in the Femtech industry.

### Review of the literature

Discourse around infertility is complex and interwoven with broader gender norms and expectations for women, mothers, and families. Several studies have noted how motherhood is often framed as a quintessential part of womanhood and women's societal value, which is used to justify the need for enduring the physical and emotional strain of pregnancy and childbirth.^11–13^ It also places considerable strain on people struggling with infertility by linking the failure to conceive to both personal and societal failures,^14^ further demonstrated by surveys in Western countries reporting that women report higher levels of distress in response to an infertility diagnosis, and are more likely than men to accept responsibility and believe they somehow brought it onto themselves.^15^

The ways in which Femtech products reinforce or challenge these narratives have been explored through interviews with users and in critical analyses of available tools, often applying Foucauldian principles to discuss the implications of tracking technologies on women and broader society. In two studies conducting interviews with women using fertility trackers to conceive in the United Kingdom, Hamper and Grenfell both found these apps influence women's sense of self and reproduction, and reinforce gender norms and neoliberal pressures on women to singularly manage reproductive responsibilities.^16,17^ These authors utilise Foucault's perspectives on power to explore the ways in which bodies are disciplined and surveilled by digital technologies, especially by biopower and biopolitics. Foucault's original descriptions of biopolitics as a technology of power focused on reproduction and fertility rates, ‘in a word, a matter of taking control of life and the biological processes of man’.^18^ Lupton references Foucault in her analysis of sexual and reproductive self-tracking apps, arguing that they position the body as both a subject and product, and surveil reproductive data to ‘impose order on otherwise disorderly or chaotic female bodies’,^19^ positioning discipline as a pressure exerted not just by governments, but also commercial and criminal actors.^20^ Sanders expands on this to argue that although digital tracking technologies are guilty of perpetuating gender roles, they also challenge institutional norms by expanding women's access to self-knowledge and care.^21^

Additionally, past qualitative work on digital fertility trackers has explored preferences and uses of cycle-tracking apps,^16,17,22–29^ built feminist frameworks to analyse tracking applications,^30–32^ and assessed privacy implications for tool use.^33–37^ Fox and Epstein's case study analysis of design choices behind menstrual tracking apps assessed app language (alongside other factors) to conclude that apps often relied on standardised experiences and did not meet users’ needs, preferences, or identities.^38^ Lupton also discusses and compares marketing taglines between sexual-tracking and fertility apps, noting the rhetoric supports reductive and normative ideas around female fertility, and calls for additional inquiry into self-tracking devices.^19^ Many authors agree that additional research investigating the influence of tracking apps, especially in the context of difficulty conceiving, is needed as these tools become more engrained into women's health management.^10,25,39–42^ While past literature has assessed app features and content, less attention has been paid to the ways that companies are advertising and marketing fertility tracking services, particularly via social media, an increasingly popular method for commercial entities and health professionals to network and connect with people trying to conceive.^43,44^

## Methods

### Aim

The aim of this research is to examine the marketing rhetoric used across fertility app websites, app store descriptions, and social media to identify thematic patterns and analyse them in the context of gendered social pressures, political changes, and medical advancements. We approached our analysis using a critical lens to assess how this advertising may both reinforce gendered aspects of reproductive labour while subverting stigmatised language around fertility and menstruation, as explored across broader areas of Femtech and self-tracking movements by Lupton and Sanders.^20,21^

### Data collection

Marketing data from a sample of 15 of the most popular infertility digital health platforms was collected to ensure sufficiently rich data content and diversity (a mix of free and paid apps, complex vs. simple data collection, data collected via Bluetooth device integration vs. manual subjective data entry, etc.). Data collection began with 10 apps and was iteratively expanded to include five more to achieve data saturation. There are no fertility-related categories on the Apple App store or Google Play store, and the list of available platforms varied between the online storefronts. Therefore most popular applications were identified using the United Kingdom Apple store search ranking (though the platforms are globally available), which weighs apps in order of popularity through downloads, quality, and quantity of ratings and reviews.^45^ The search term used was ‘Fertility Tracker’, based on past audits of fertility tracking applications on the Apple Stores that found the permutation of ‘Fertility Tracker’ produces the largest number of apps, and most accurate selection.^46^

Since this research is focused on platforms that are replacing or supplementing traditional fertility pathways, inclusion criteria were developed to focus the data collection on more robust platforms:

Inclusion criteria (date of inclusion 06/2023):

- App includes a robust fertility tracking feature for humans
- App is based on cited scientific research, formal efficacy testing, or feedback from medical advisors
- App is currently functioning and has not been retired
- App on the Apple Store

This still resulted in a diverse set of platforms of varying quality, sophistication, lifespan, and user populations. The marketing claims from platform websites, App store pages, three posts on their Facebook pages, and transcription of their introductory YouTube Channel videos (if available) were collected for analysis through June and July of 2023. Most of this data was text-based, and video content was transcribed into text for analysis. Images were also collected when context was needed to interpret text excerpts. These platforms have been identified in past research as social media platforms women use for health information, health advice, and support, and were the most common social media platforms on which the 15 selected apps had presence.^47^ Though other platforms, including Instagram, TikTok, and X (formerly Twitter) are popular among female users, they were used inconsistently by the sampled apps and were excluded to provide a more uniform structure to the data.

Ethical and privacy considerations were made by the authors while collecting app advertising content to ensure social media data was kept anonymous, and did not publicise private individual's identities or risk sharing sensitive or potentially embarrassing individual data.^48^ All marketing materials analysed were published online and were made for and widely viewed by people using social media and the app store. All materials were extracted from public-facing company sites and social media pages intended for general consumption, and no content was collected from people's personal social media pages. Thus, since these materials were in the public domain, additional permissions were not sought to view or analyse company marketing content.

### Thematic coding

Marketing data from the platforms was collected and centralised in a spreadsheet for analysis summarised below in Table 1 (*n* = 105) during June and July 2023. The spreadsheet also included a column for coding, general notes, and notes on outlier or miscellaneous data.

**Table 1.** Data sources.

Data Source	Text Excerpts
Platform Website	33
App Store Description	15
YouTube Channel	12
Facebook Page Posts	45

Braun and Clarke's method of inductive thematic analysis was used to familiarise ourselves with the collected marketing data, identify initial codes from patterns across the data set, and collate initial codes into themes with discussion with the research team from August to November 2024.^49^ The coding set was developed inductively following a ‘data to theme to theory’ approach to identify themes from the data rather than fit them into an existing framework.^50^ The flexibility of this approach was helpful in studying the area of digital fertility tracking, given that research in the area is still emerging.^51^ To clarify codes and reduce risk of overlap, all codes were tracked and defined through a spreadsheet template. Close attention was paid to the language used by each company to describe human and non-human elements, how norms were perpetuated or challenged, and how fertility tracking behaviours were framed. The whole data set was re-reviewed to ensure inclusion of any material missed during the initial coding process.

## Results

**Figure 1.**
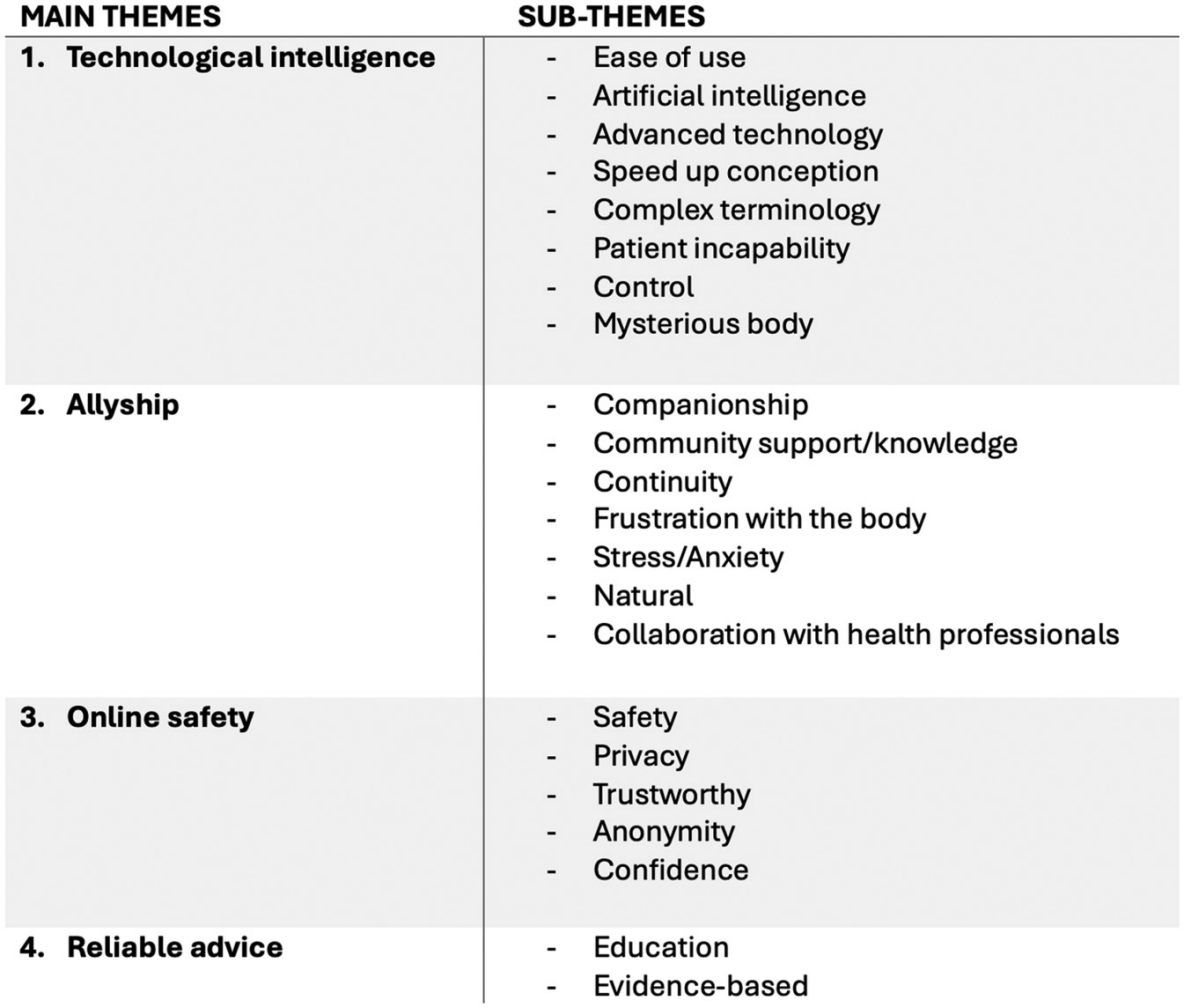
Thematic coding tree.

The platforms analysed varied from relatively simple digitised ovulation predictors to highly sophisticated, AI-powered platforms with integrated telehealth and self-collected lab testing. Most of the apps selected were free to download and use at a very basic level, with subscriptions or in-app purchases available to access enhanced user features. Several platforms could be used (and are perhaps better known) as menstrual trackers or contraception aids and then switched to a ‘conception’ setting when trying to conceive. Most apps also offered customisable data visualisation, which ranged from simple calendar views highlighting potentially fertile days to interactive longitudinal symptom graphs and cycle histories. In addition to this, many platforms included educational pages to help users interpret their data and community pages where users could connect and share health information or offer support. Most platforms had an international presence with large followings across English-speaking countries in Europe, the United States, and Australia. After consolidating similar codes, we identified four major themes across app marketing explored below in order of frequency: an emphasis on technological rather than human intelligence, allyship, online safety, and reliable advice (see Figure 1).

### Emphasis on algorithms and technology over human intelligence

The most common theme, ‘Emphasis on Algorithms and Technology’ frames knowledge derived from artificial intelligence or advanced technology as more beneficial than that from people (e.g., self, family, or friends) in the user's own social world. This marketing could be perceived as overtly underestimating the abilities of women in interpreting test results, such as ‘interpret LH test strips with the app, not your eyes!’^52^ but mainly suggested their ‘big data libraries’ and ‘machine learning algorithms’ were more effective than human awareness and organisational capacity. These marketing statements often included dense technical language perhaps to communicate their complexity:

In-Depth Analysis of Your Fertility Data: The Pregnancy Monitor scrutinises your data for early signs of pregnancy and displays a summary of its findings: A reliable test date. An analysis of your intercourse timing. A statistical analysis of your signs against our extensive database. A triphasic pattern detector. An analysis of luteal phase spotting. Data on pregnancy testing from our test database. A more accurate due date taking your cycle characteristics into account.^53^

A few of these statements suggested that the platform was more effective than clinical care, with quotes like ‘Do yourself a favour and make the investment [in the app] before throwing money at doctors who cannot give you the amount of information [the app] can’.^54^ This runs counter to many of the risks introduced by algorithm-based ovulation calculators, which have been shown to miss or incorrectly calculate fertility windows by standardising users’ unique fertility journeys, resulting in low accuracy and quality.^55–57^ These algorithms frequently rely on measurements based on ‘average’ ovulation cycles that can be influenced by lifestyle or health factors, such as basal body temperature (BBT), which can vary based on waking time, quality of sleep, and alcohol consumption, or luteal hormone detection, which can be affected by fever or conditions like polycystic ovarian syndrome.^58^ In contrast to platform claims, interviews with reproductive endocrinology and infertility clinicians show this is often why clinicians are hesitant to trust patient-generated fertility tracking data and prefer to generate their own highly personalised radiology, laboratory, and clinical medical history data.^59^

References to ‘big data’ and artificial intelligence emphasise reproductive bodies as subjects that can be measured and monitored best through technology, and therefore finally understood and controlled. Interpreting these home technologies introduces its own challenges, particularly when results do not include a standardised reference value or the confidence interval; some apps emphasised that compared to typical ovulation tests, theirs were easier to interpret and work:

You may be confused when receiving continuous positive results … Instead of giving a positive or negative result, quantitative ovulation test alone give a numerical LH level range from 2.5 to 80 miuml.^60^

Several marketing statements also focused on speed to conception, suggesting with little evidence that conception would be faster with their data tracking method: ‘Ready to reach your fertility goals faster? Try [app] today’.^61^ In other cases, companies used numerical data to claim their special ‘predictive intelligence’ was faster, without citing any relevant research or information to back up their claims: ‘Women who rely on our predictive intelligence to calculate their ovulation day get pregnant on average in 58 days. That's 123 days faster than the national average’.^62^ The lack of context around this ‘national average’, despite the company's tagline of ‘Because Accuracy Matters’, makes it very difficult to interpret or generalise, though it may be based on a commonly cited reference that 80% of couples conceive within 6 months (180 days) of starting to try and conceive.^62,63^ Similarly, claims such as ‘Research shows that couples get pregnant in three cycles or less on average with [app] … this is because the [app] algorithm uses your temperature data to find your unique fertile window so you can time sex and conceive faster’, referencing research the company funded, again may give users the impression their time to conception will be quickened by use of BBT, which as discussed above, may poorly predict ovulation depending on lifestyle factors and quality.^58,64^

### Allyship

The next theme, ‘Allyship’, suggested that users would have a companion with knowledge and empathy, either through the platform's support communities, or medical professionals. Framed as a constant supporter, these platforms offered continuity of care not just through fertility tracking, but all the way through pregnancy and post-partum, with quotes like ‘with you through it all … one companion for life’^65^ and ‘[App's] goal is to empower females to take control of their health and prioritise their well-being at all stages of their life’.^66^ Some also touched on their role in the workplace along with home use if offered as a workplace benefit; ‘The company empowers women to take control of their healthcare both at home and at work’.^67^

Virtually all of the platforms specifically targeted women or femininity in their marketing, and though a few promoted a ‘couples’ or ‘partner’ option, most of the tracking was still specific to female biology. Users were also typically portrayed as women, such as using high-pitched, stereotypically feminine exclamations pretending to discover a mobile phone app; ‘What's this on my phone … well … yes? Oh my gosh! Where am I now? Wow, look at all these pregnant women!’.^68^ Others showed comradery by referencing shared experiences in the challenges of having unpredictable cycle dates (which can make trying to conceive using time-based intercourse challenging) with feminised language; ‘Does your period have a flair for the dramatic, always arriving fashionably late or overstaying its welcome … But guess what? Irregular cycles don't mean you can't conceive!… That's right, even if you have irregular cycles, the next success story could be yours’.^69^ Becoming one of the ‘success stories’ of women who finally had ‘control’ over their health and could understand how their body and menstrual cycle ‘works’ was a common thread across company pages.

Others emphasised allyship, community belonging, and the importance of not experiencing infertility alone. They often emphasised how their brand was not just a piece of technology, but a friend, or a way of making new friends with the common interest of trying to conceive. The allyship marketing for fertility apps acknowledged the difficulties and isolation of infertility and offered soothing support, free of stigma:

Self discovery without shame.^65^

Get a second opinion on your charts from fellow charting or fertility awareness-based method enthusiasts, find support from people on the same journey as you, and maybe even forge lasting friendships in our global [app] community. [App name] is more than just an app.^70^

We don’t know what we don’t know, and that's why we built the [app] Community so there was a safe place to ask questions without judgement. (And vent without judgement too.) Join the community.^71^

These platforms sometimes featured embodied ‘little helpers’, such as one platform with an ‘AI assistant’ named ‘lollipop’, giving them high-pitched, childish voices to make them seem friendly.^68^

The language used in the allyship theme includes platforms that frame their role as providing access to communities of support (rather than just an ovulation calendar) alongside education and supportive guidance: ‘[app name] are more than just trackers. They’re a supportive community of women who are on the same journey as you’.^72^ Several touched on the difficulties of navigating the fertility industry and various steps in clinical care and advertised telehealth services to help with the process, or special data report formats that could be brought to doctors’ appointments:

It's like having an endocrinologist on call (hormones! science! help!) – for free.^73^

Like having a healthcare professional in your pocket, ready to guide your journey to motherhood and wellness.^72^

Make [app] your reliable health care partner with insights overview. Perfect for any doctors visit!^74^

Email and/or print your charts to share with your doctor, friends, or family.^75^

In some cases, they adopted a medicalised lens to present the app as a clinical aide, visually guiding the patient towards virtual providers representing the app. However, given risk of health misinformation online, several tools presented their online communities carefully, highlighting fact-checking posts:

There are all sorts of ideas, practices, and advice to members of the TTC [trying to conceive] community. Here are a few common ones you may have heard, and if there's any facts behind them.^76^

### Online safety

This theme includes marketing claims that platform users’ data will be protected from being sold or provided upon request to government or commercial institutions. The concern in these posts notably increases following the overturning of Roe V. Wade, which ended the constitutional right to abortion in the United States and introduced major concerns that the reproductive data used by fertility tracking apps could be used to incriminate women suspected of having an abortion. Several platforms responded to user concerns over their data safety on social media citing Roe V. Wade or differentiated themselves as companies that supported the pro-choice movement:

In a post Roe v Wade world, we've had a lot of questions from our users about how we keep their data safe in [app name]. Today we're sharing how we've prioritized privacy since we launched in 2012 and how we'll continue to ensure our users’ data is secure and anonymous in this evolving world.

We trust women, and that means supporting a woman's right to choose. We’re extending our employee donation matching policy to our community by matching all of your donations to Planned Parenthood … We’re in this together.^71^

Though the fertility apps included in this sample are available internationally and several were created by corporations outside of the United States, the size of the American investment and user market would be likely to have influenced companies to acknowledge implications of the legislative changes, exemplified by this European-based app:

As the team behind [app name], we have cycles, periods, and pregnancies, and we track them in the app. There is simply no way we’d *ever* hand over private health data to anyone who would use it against you. And we would let you know if anyone ever tries.^77^

The increased liability of reproductive data in the United States shone a spotlight on just how much data were being collected by these platforms, who had access, and whether it could be used to incriminate women. Apps incorporated in the United States that may not be able to guarantee they would withhold user data under a court subpoena introduced ‘Anonymous Mode’ settings which a user could select to keep their identity private. In anonymous mode, one app stated that ‘in light of Roe v. Wade being recently overturned’ a free option would be available to users to remove identifying information from their profiles, so that ‘In the event that [app company] receives an official request to identify a user by name or email’ they would be unable to comply.^78^ Some directly refer to the predatory data mining practices of their competitors, and the risks associated with them;

You are [app's] customer and our loyalty is to YOU. If you do not know how a ‘totally free’ App/Service is funded, or if you know it is funded with ads, referrals, or your data, you are likely the product that is sold, not the customer.^53^

Total privacy: We take your privacy very seriously! Unlike many other women's health apps, we don't require your email address to sign up, we don't sell or share your personal health data. It's your life and it should stay with you!^79^

### Reliable advice

A portion of platforms claimed to deliver high-quality, research-based information on fertility and/or conception, purposely contrasting themselves from apps that essentially just digitised the ‘natural family planning’ method, a basic way of tracking menstruation to identify more fertile phases to plan or prevent pregnancy. The technology behind these platforms was often more advanced, designed with medical professionals, or under guidance from a medical board. Several advertised clinical-level testing and claimed that unlike other platforms, they were research-based and science-driven: ‘Built on empirical evidence and a culture of science, [app] has shown measurable impact on family health outcomes, preventing unnecessary medical costs’.^80^ Some platforms prioritised evidence of these claims on their websites, with prominent posts exhibiting published research on their tools or research collaborations, or offering for users to become investors in their apps to support research:

There's no question that menstrual and reproductive health don't get the funding, research, or innovation that's needed. But we're ready to change that–together … when you become a co-owner … you'll be closer than ever to our product development process, helping to shape the future of the app, and supporting important research in this space.^77^

These advertisements often used more scientific and health jargon instead of lay terms, possibly to differentiate the platform from competitors:

Did you know scientists use fluorescence-based tech at the fertility lab? So does [app name]. We use immunochromatography with fluorescence labelling.^81^

It is worth noting that though many platforms commonly referred to themselves as ‘research-based’, only a few provided tangible information to users about their design process, published results, or source of educational materials. Several tools with highly medicalised branding and ‘lab-grade’ quality tests simultaneously advertised ‘fertility-boosting’ teas and supplements unsupported by research, or online coaching and meditation classes with ‘experts’ that had gained certification through the app's $900 ten-week class covering topics such as anatomy and medical management:

The [app name] can help you make sense of these symptoms … Check out our online or in-person classes with a certified [app] teacher.^73^

## Discussion

This work identifies the rhetoric used in four key themes present across the marketing of 15 top fertility tracking applications available for smartphones and used by millions across the globe. These themes emerge during an interesting moment for Femtech, amid (in some cases, tense) political, social, and cultural changes in reproductive health, and indicate that attitudes around using digital technologies for decision-making and companionship have changed in the area of fertility.

Until recently, a company marketing their tracking algorithms as more (or as) effective compared to a doctor or medical professional would have been unusual, but the emergence of ChatGPT has radically improved the capabilities of predictive artificial intelligence and personality of chatbots. The emphasis on non-human elements, particularly artificial intelligence, seen in the ‘Algorithms’ theme alongside the empathic and humanistic framing of apps in the ‘Allyship’ theme also reflect the widespread adoption and normalisation of AI in consumer-facing technologies. The marketing in the ‘Algorithms’ theme evidences the difficulty in discerning what company data are accurate, and how easily attractive marketing claims can instil false expectations and hope. In a society where 25% of women with fertility challenges face unexplainable infertility, this language also tempts users with the biopolitics of hope, previously explored by Mayes, Williams, and Lipworth in relation to the egg freezing industry and Perrotta and Hamper to discuss the appeal of IVF add-ons.^82–84^ In the context of ovulation tracking, it intertwines hope with the responsibility of conception and frames reproduction as something that can be configured or wrangled by women using a company's digital technologies. This responsibility and hope may be misplaced. Blanket claims of reducing time to conception, for example, overlook the complexity of individualised fertility predictions. For example, average time to conception increases for women over 40, and tracking may have a different impact on those who are obese or exposed to certain lifestyle and environmental factors like smoking, stress, or alcohol, which can decrease chances of conception.^85^ Oversimplified marketing claims also fail to acknowledge common biological causes of sub- or infertility that cannot be addressed through menstrual tracking, such as ovulatory disorders (e.g., luteal phase defects, polycystic ovary syndrome), or male factor infertility.^86^

Similarly, the ‘Allyship’ theme marks a shift in the availability and popularity of digitally mediated relationships. Past assessments of social support for infertile women typically mention the individual's family, friends, or significant others, while these marketing statements promoted the phone as a companion, or as the connection point to community.^87,88^ Technology as a source of comfort was evident in language that framed apps as a safe space and acknowledged the challenges and pains of womanhood. Though the process of turning symptoms into data does not impact their severity or frequency, tracking is frequently presented as an improvement to women's fertility management throughout the data. Other research supports this finding; Hamper's interviews with users of fertility trackers found that the work participants invested in tracking their symptoms intensified the reproductive process to control and conceptualise otherwise ‘invisible’ reproductive symptoms and labour.^17^ However, companies framing their tracking platforms as constant support, particularly while using medicalised language, risks further individualising the management of fertility journeys by shifting responsibility away from established healthcare systems and towards users. Though ChatGPT-based chatbot integration in some platforms may hold promise for delivering information and support for infertility patients, current models still fall short of the level of ‘community’ and nuanced support traditional social structures provide.^89,90^

This language reflects the serious unmet needs in the provision of fertility care; the experiences of loneliness, isolation, and stigma common among those facing infertility paired with the convenient access and anonymity offered by mobile-based applications perhaps explains why these apps can offer such intimate communal support to their users, also reflected in Grenfell's findings.^16^ These statements in some ways challenge social norms which treat menstruation and infertility as something that should be concealed, particularly compared to language around fertility which individuals with infertility report can make them feel blamed or reinforce stigma.^91^ The popularity of community forums across platforms and emphasis on offering constant, caring support raises questions about the psychosocial support these platforms may offer users, and how this may influence delivery of (mis)information. The tension between the themes identified in this work is continually renegotiated across the marketing sample; the same platforms that offer women support and emphasise trust in the ‘Allyship’ theme simultaneously grapple with the oppressive power and potential threats posed by governments and tech giants through user privacy and data ownership, as explored in the ‘Safety’ theme.

The language used between different platforms (or even within the same platform) could be disorienting, as seen in the ‘Safety’ themes, where companies jostled against each other to differentiate themselves as a truly trustworthy or evidence-based app. The theme of ‘Safety’ is noteworthy given several companies, even those not based in the United States, made prominent social media posts or introduced new anonymous features addressing privacy and security concerns following the overturning of Roe v. Wade. Some posts openly acknowledged fear of state surveillance and discipline, while others hinted at surveillance from the marketing and advertising industry more broadly, reflecting the pressures from both government and commercial actors as discussed by Lupton.^20^ This perhaps explains the overtly pro-abortion posts from several companies, a striking decision given the taboo of abortion in marketing.

Together, these themes point to the rapid diffusion of digital trackers into the lifestyle and well-being space, presenting as technically and medically sophisticated sources of emotional support and community. Our findings have implications for patients, providers, and policymakers in the reproductive health space. Patients should be cautious of marketing language that promote scientific claims without a clear citation or explanation, and check whether medicalised service provisions in an app are provided by health professionals who are certified through nationally regulated certification bodies, if this is what they seek. Providers should be aware that these apps may be much more than ‘just an app’ to patients, given the degree of psychosocial language in the ‘allyship’ theme and frequency of private telehealth integration within paid applications. Policymakers should note the murky market that patients must navigate when seeking a fertility tracking app, perpetuated by the lax regulation of marketing claims, highlighted by other authors.^92^ The prevalence of incomplete or problematically presented ‘data’ and ‘research’ across app marketing, and the encroachment of non-clinical fertility ‘coaches’ as health professionals capable of providing medical guidance may give users an inaccurate impression of the scientific rigour behind these platforms, or risk exposing them to health misinformation.

### Limitations and future research

There are significant differences across social media platform algorithms, usability, user bases, and watch times, which limits the applicability of these outcomes to different social media content, or content not produced in English. Consequently, these findings may not be applicable to all global technology landscapes, and additional research may explore and compare how companies advertise in non-English-speaking contexts. It would be particularly interesting to compare how companies advertise on social media platforms most popular with younger demographics, especially in relation to advertising that highlights anxieties around (in)fertility and declining fertility rates.

As the use and examination of fertility tracking platforms increases, differentiating between types of tools will become more important, as noted by both researchers and developers.^28,93^ This work contributes by identifying major themes emerging from the self-tracking and fertility industry to capture this variability across the market through a critical lens and explore the resulting tensions. It also discusses the implications this rhetoric may have for patients, providers, and policymakers. This approach cannot show how their intended audience perceives or responds to the marketing strategies, nor whether and how the strategy influenced users’ experience with the apps. However, these findings provide an interesting comparison to user's interpretations of in-app language and add further evidence of the ways corporations entrench disciplinary language and framing of (in)fertility.

## Conclusion

Despite being a cornerstone of reproductive justice, fertility services remain out of reach for many worldwide due to stigma, social barriers, eligibility criteria, and cost. Digital fertility management platforms may offer an accessible and promising disruption to the existing industry. However, this analysis shows that the marketing used to sell these apps claims to be expanding user's access to medical information and community support without acknowledging the consequential responsibilities introduced by self-surveillance. Some of the language used is overt, such as in the case of messaging that encourages women not to trust their own bodies and compares the intelligence of digital trackers to specialist medical professionals. Subtler regulations and expectations of social order also impart harmful messaging of gendered pressures and responsibility. When this disciplinary language is paired with paid subscription services or the harvesting and selling of user data, these tools risk perpetuating the same issues they hope to address.

Though there are some examples of subversive language combatting social silence and taboo around infertility and menstruation, the overall messaging still fulfils stereotypes around how women should be treated and viewed during their cycle and while trying to conceive. Yet changes to reproductive rights worldwide may be giving Femtech a push towards challenging institutional repression more boldly, as seen by platforms’ pro-abortion rhetoric in response to abortion restrictions in the United States, and in efforts to bring digital services to underserved areas to promote access to women's health education. As patient-facing technologies become further entrenched in fertility care, it will be interesting to see whether the framing of digital tools as empowering ways to *avoid* clinical care, or conversely as means to *access* trustworthy clinical care will endure. Moreover, whether increasing availability of app-based health education, community, and tracking may challenge current models of fertility care.
